# Genome-Wide Gene Expression Profiling Defines the Mechanism of Anticancer Effect of Colorectal Cancer Cell-Derived Conditioned Medium on Acute Myeloid Leukemia

**DOI:** 10.3390/genes13050883

**Published:** 2022-05-15

**Authors:** Ji-Eun Lee, Chan-Seong Kwon, Byeol-Eun Jeon, Woo Ryung Kim, Du Hyeong Lee, Sara Koh, Heui-Soo Kim, Sang-Woo Kim

**Affiliations:** 1 Department of Integrated Biological Science, Pusan National University, Busan 46241, Korea; dlwldms4535@naver.com (J.-E.L.); ckstjd5091@naver.com (C.-S.K.); starsliver20@naver.com (B.-E.J.); dnfud647@pusan.ac.kr (W.R.K.); doo2080@naver.com (D.H.L.); khs307@pusan.ac.kr (H.-S.K.); 2 Department of Biological Sciences, Southern Methodist University, Dallas, TX 75206, USA; kohs@mail.smu.edu; 3 Department of Biological sciences, Pusan National University, Busan 46241, Korea

**Keywords:** gene expression profiling, acute myeloid leukemia (AML), conditioned medium, anticancer effect

## Abstract

Acute myeloid leukemia (AML) is the most common type of leukemia in adults, accounting for 30% of all adult leukemia cases. While there have been recent improvements in the prognosis of the disease, the prognosis remains grim, and further understanding of AML and the development of new therapeutic agents is critical. This study aimed to investigate the potential interaction between colorectal cancer (CRC) cells and AML cells. Unexpectedly, we found that CRC cell-derived conditioned medium (CM) showed anticancer activities in AML cells by inducing apoptosis and differentiation. Mechanistic studies suggest that these phenotypes are closely associated with the suppression of PI3K/AKT/mTOR and MAPK survival signaling, the upregulation of myeloid differentiation-promoting transcription factors *c/EBPα* and *PU.1*, and the augmentation of executioner caspases-3/7. Importantly, bioinformatic analyses of our gene expression profiling data, including that derived from principal component analysis (PCA), volcano plots, boxplots, heat maps, kyoto encyclopedia of genes and genomes (KEGG) pathways, and receiver operating characteristic (ROC) curves, which evaluate gene expression profiling data, provided deeper insight into the mechanism in which CRC-CM broadly modulates apoptosis-, cell cycle arrest-, and differentiation-related gene expression, such as *BMF*, *PLSCR3*, *CDKN1C*, and *ID2,* among others, revealing the genes that exert anticancer effects in AML cells at the genomic level. Collectively, our data suggest that it may be worthwhile to isolate and identify the molecules with tumor-suppressive effects in the CM, which may help to improve the prognosis of patients with AML.

## 1. Introduction

Hematopoietic stem cells (HSCs) are differentiated into myeloid lineage cells and lymphoid lineage cells, which are further differentiated into a variety of different blood cells. Lymphoid B cells, T cells, and NK cells are derived from lymphoid lineage progenitors, while erythrocytes, megakaryocytes, granulocytes, macrophages, mast cells, and dendritic cells are derived from myeloid lineage progenitors [1,2]. Myeloid cells are involved in oxygen transport, blood clotting, inflammation, and innate immune response [3,4]. Common myeloid progenitors (CMP) derived from hematopoietic stem cells can differentiate all myeloid lineage cells. The formation of various blood cell types from progenitor cells is a complex process, involving different cytokines. For example, hematopoietic cytokines, such as granulocyte-colony-stimulating factor (G-CSF) and erythropoietin (EPO), regulate the differentiation of common myeloid progenitors (CMPs) into different types of myeloid cells. The binding of these hematopoietic cytokines to their cognate receptors induces myeloid progenitor survival and myeloid differentiation by activating transcription factors, including PU.1, CCCAT/enhancer proteins, interferon-regulatory factor 8 (IRF8), and runt-related transcription factor 1 (RUNX1). PU.1 is one of the transcription factors that induces differentiation into monocytes. Unsurprisingly, diseases such as acute myeloid leukemia (AML) are associated with the dysregulation of myeloid differentiation [4,5,6,7].

AML is the most common type of acute leukemia in adults and is characterized by the clonal expansion of hematopoietic progenitor cells that fail to differentiate normally in bone marrow and peripheral blood [8]. The incidence of AML increases with age. In 2021, approximately 20,240 new cases of AML and 11,400 deaths were reported worldwide [9,10]. Chromosomal abnormalities and mutations affecting hematopoietic cell proliferation and differentiation have been frequently found in AML patients. [10,11]. Mutations in *FLT3* promote the proliferation and survival of blasts by activating the Ras-Raf-MEK-ERK, JAK-STAT, and PI3K-AKT signaling pathway. In addition, mutations in *RUNX1* or *c/EBPα* stimulate leukemogenesis by inhibiting differentiation into specific myeloid lineages [11,12]. Furthermore, *AML1-ETO* and *PML/RARα* fusion genes that result from t (8;21) and t (15;17), respectively, impair myeloid differentiation and potentially contribute to the development of AML [13,14]. The standard treatments for AML consist of the combination of an anthracycline and cytarabine, followed by consolidation chemotherapy, or allogeneic stem cell transplantation. Currently, approximately 40% of AML patients aged 60 years or younger, and only about 10% of patients aged 60 years or older are cured [15,16]. Thus, there is a need to develop a better understanding of this disease and the development of new therapeutic agents.

The secretome constitutes the set of proteins secreted by cells into the extracellular matrix and includes growth factors, cytokines, chemokines, enzymes, and hormones. These factors, secreted individually or in vesicles, affect cells or tissues in an autocrine, paracrine, or endocrine manner. The secretome plays an important role in biological processes, such as immune defense, cell signaling, and cell–cell communication [17,18]. It is also involved in cell apoptosis; adipose-derived stem cell conditioned medium decreased cisplatin-induced cell apoptosis in the tongue squamous cell carcinoma cell lines, SCC-25 and CAL-27. Cell culture medium, called “conditioned medium”, contains various factors secreted by cells, such as secretome, microvesicles, or exosome [19]. The anti-apoptotic effect of adipose-derived stem cell conditioned medium was induced via the elevation of IGF-1R/AKT/ERK signaling. Adipose-derived stem cell conditioned medium also alleviated cisplatin-triggered apoptosis by increasing pro-caspase-3, pro-caspase-9, and phospho-BAD expression levels [20]. In addition, recent studies have shown that the secretome affects cell differentiation. Mesenchymal stromal cell-derived secretome inhibited myofibroblast differentiation. As fibrosis processes, fibroblasts differentiate into myofibroblasts, secreting extracellular matrix proteins. Expression of α-SMA and collagen type I increases during fibrosis, but the secretome from mesenchymal stromal cells inhibits their expression. Furthermore, mesenchymal stromal cell-derived secretome induced the de-differentiation of myofibroblasts into fibroblasts [21].

Intriguingly, Sawa-Wejksza et al., have shown that CRC-CM induced differentiation of THP-1 acute monocytic leukemia cells into macrophages, leading to decrease in the proliferation and cell cycle arrest at the G1 stage. Although the underlying mechanism is unclear, these results suggest that CRC-CM can potentially be used as an agent to inhibit the proliferation of AML cells [22]. In this study, we investigated the potential effect of CRC-CM on apoptosis and the differentiation of AML cells.

## 2. Materials and Methods

### 2.1. Cell Culture and Antibodies

Human AML cell lines (KG1 and HL-60) and human colorectal cancer cell lines (DLD1 and HCT116) were obtained from the Korean Cell Line Bank (Seoul, South Korea). KG1, HL-60, DLD1, and HCT116 cells were cultured in RPMI-1640 Medium (Cytiva, SH30027.01, Marlborough, MA, USA), supplemented with 10% fetal bovine serum (FBS; Capricorn Scientific, FBS-12A, Ebsdorfergrund, Germany), 1% L-glutamine (Gibco, 25030-81, Waltham, MA, USA), 1% N-2- hydroxyethylpiperazine-N’-2-ethanesulfonic acid (HEPES; Gibco, 15630-080) buffer, and 1% penicillin/streptomycin (PEN/STR; Gibco, 15140-122) at 37 °C in a 5% CO_2_ incubator. The primary antibodies used in this study were anti-β-actin (1:5000 dilution; sc-47778; Santa Cruz Biotechnology, Dallas, TX, USA), anti-pAKT (1:1000 dilution; 9271S; Cell Signaling Technology, Beverly, MA, USA), anti-p4EBP1 (1:5000 dilution; 9459S; Cell Signaling Technology, Beverly, MA, USA), and anti-pERK1/2 (1:1000 dilution; 9101S; Cell Signaling Technology, Beverly, MA, USA).

### 2.2. Preparation of Conditioned Medium

DLD1 and HCT116 colorectal cancer cells (2.0 × 10^6^ cells per well) were seeded in 6-well plates. After 48 h, the culture medium was collected to be used directly or stored at −80 °C for later use. Conditioned medium (CM) derived from KG1 cells was used to control for the possible decrease of growth and survival factors in the CRC-CM. In other words, 20% DLD1 CM contains 20% DLD1 CM plus 80% KG1 CM, and 50% DLD1 CM contains 50% DLD1 CM plus 50% KG1 CM.

### 2.3. Measurement of Cell Viability

The CellTiter 96 Aqueous MTS assay was used to identify the cytotoxicity of CRC-CM. KG1 and HL-60 cells were seeded at 3.0 × 10^4^ cells per well in 96-well cell culture microplates and treated with DLD1- or HCT116-derived CM for 48 h and 72 h. Then, 30 μL of MTS reagent (Promega, G1112) was added to the wells and incubated at 37 °C for 2 h. Absorbance was measured at 450 nm using a GloMax^TM^ Microplate multi-mode reader (Promega, Madison, WI, USA). 

### 2.4. Trypan Blue Staining

KG1 and HL-60 cells were seeded at 2.0 × 10^5^ cells per well in 24-well plates and treated with DLD1- or HCT116-derived CM for 72 h and 96 h. The cell suspension was then mixed 1:1 with 0.4% trypan blue stain (Gibco; 15250061) and stained for 3 min at room temperature. The positively stained cells were counted using a hemocytometer (Marienfeld, Lauda-Königshofen, Germany) under a phase-contrast microscope (Olympus CKX41, Olympus Corporation, Tokyo, Japan).

### 2.5. Giemsa Staining

Cells were fixed for 7 min in methanol, air dried, and stained for 15 min with Giemsa stain (Sigma, 48900-500ML-F, St. Louis, MO, USA). Cells were then rinsed with deionized water and air-dried. The cells were imaged with an Olympus CX31 microscope (Olympus Corporation, Tokyo, Japan) at 400× magnification. Representative images were captured using Images Plus 2.0 software (Motic Co., Ltd., Xiamen, China). 

### 2.6. Quantitative Real-Time RT-PCR (qRT-PCR)

The transcription levels of *c/EBPα, PU.1, ITGAM, BIRC5, BMF, MYB, NDRG1, RBM38, CDKN1C, MKI67,* and *PLK1* were measured by qRT-PCR. RNA was extracted using TRIzol reagent (Favorgen, FATRR 001, Wien, Austria). PrimeScript RT reagent Kit (Takara, RR047A, Kusatsu-shi, Japan) was used for cDNA synthesis. Real-time qRT-PCR was conducted using TOPreal qPCR PreMIX SYBR Green with low ROX (Enzynomics, RT500M) [23]. The sequences of the primers are listed below: 

c/EBPα Forward: 5′–AACCTTGTGCCTTGGAAATG-3′

c/EBPα Reverse: 5′–CCCTATGTTTCCACCCCTTT-3′

PU.1 Forward: 5′–ATGTGCCTCCAGTACCCATC-3′

PU.1 Reverse: 5′–CAGGTCCAACAGGAACTGGT-3′

ITGAM Forward: 5′–AGAACAACATGCCCAGAACC-3′

ITGAM Reverse: 5′–GCGGTCCCATATGACAGTCT-3′

BIRC5 Forward: 5′–GCCTTTCCTTAAAGGCCATC-3′

BIRC5 Reverse: 5′–AACCCTTCCCAGACTCCACT-3′

BMF Forward: 5′–CTGGGAAGTGGACTGTGGTT-3′

BMF Reverse: 5′–GGCAGGTACTGGCTGAGAAG-3′

MYB Forward: 5′–GGCAGAAATCGCAAAGCTAC-3′

MYB Reverse: 5′–GCAGGGAGTTGAGCTGTAGG-3′

NDRG1 Forward: 5′–ACAACCCTGAGATGGTGGAG-3′

NDRG1 Reverse: 5′–TGTGGACCACTTCCACGTTA-3′ 

RBM38 Forward: 5′–AGAAGGACACCACGTTCACC-3′

RBM38 Reverse: 5′–GTCTTTGCAAGCCCTCTCAG-3′

CDKN1C Forward: 5′–TGCACGAGAAGGTACACTGG-3′

CDKN1C Reverse: 5′–GTGCCTTTGGCATAACCAAT-3′

MKI67 Forward: 5′–AAGCCCTCCAGCTCCTAGTC-3′

MKI67 Reverse: 5′–TCCGAAGCACCACTTCTTCT-3′

PLK1 Forward: 5′–AAGAGATCCCGGAGGTCCTA-3′

PLK1 Reverse: 5′–GCTGCGGTGAATGGATATTT-3′

TBP Forward: 5′–TATAATCCCAAGCGGTTTGCTGCG-3′

TBP Reverse: 5′–AATTGTTGGTGGGTGAGCACAAGG-3′

### 2.7. Western Blot Analysis

To perform Western blot analysis, KG1 and HL-60 cells were seeded in a 12-well plate at 1.0 × 10^6^ cells per well and were treated with DLD1- or HCT116-derived CM for 48 h, followed by harvest and lysis in RIPA buffer (ELPIS Biotechnology, EBA-1149, Daejeon, Korea) with 1 mM Na-vanadate (Sigma), 50 mM β-glycerophosphate disodium salt (Sigma, G9422), β-mercaptomethanol (142 mM; BioWORLD, 41300000-1), Protease Inhibitor Cocktail (Sigma, P8340), and EDTA (5 mM; Thermo Scientific, 78441). Samples were boiled at 100 °C for 10 min in sample buffer, loaded in the polyacrylamide gels, and transferred onto Immobilon-P Transfer membranes, followed by blocking in 1% bovine serum albumin (BSA; MP Biomedicals, 160069, Irvine, CA, USA). The membranes were incubated with primary antibodies at 4 °C overnight. After washing 3 times with Tris-buffered saline containing Tween 20 (TBST) for 5 min each, the membranes were incubated with anti-mouse secondary antibodies (Santa Cruz Biotechnology, sc-516102, Dallas, TX, USA) or anti-rabbit secondary antibodies (BETHYL, A120-101P, Hamburg, Germany) for 1 h at room temperature. After washing 3 times with TBST for 10 min each, the membranes were exposed to a chemiluminescent substrate (EzWestLumi plus (ATTO, WSE-7120L), and protein bands were identified using the Luminograph II (ATTO, Osaka, Japan) [24]. 

To perform Western blot analysis, KG1 and HL-60 cells were seeded in a 12-well plate at 1.0 × 10^6^ cells per well and were treated with DLD1- or HCT116-derived CM for 48 h, followed by harvest and lysis in RIPA buffer (ELPIS Biotechnology, EBA-1149, Daejeon, Korea) with 1 mM Na-vanadate (Sigma), 50 mM β-glycerophosphate disodium salt (Sigma, G9422), β-mercaptomethanol (142 mM; BioWORLD, 41300000-1), Protease Inhibitor Cocktail (Sigma, P8340), and EDTA (5 mM; Thermo Scientific, 78441, Waltham, MA, USA). Samples were boiled at 100 °C for 10 min in sample buffer, loaded in the polyacrylamide gels, and transferred onto Immobilon-P Transfer membranes, followed by blocking in 1% bovine serum albumin (BSA; MP Biomedicals, 160069). The membranes were incubated with primary antibodies at 4 °C overnight. After washing 3 times with Tris-buffered saline containing Tween 20 (TBST) for 5 min each, the membranes were incubated with anti-mouse secondary antibodies (Santa Cruz Biotechnology, sc-516102) or anti-rabbit secondary antibodies (BETHYL, A120-101P) for 1 h at room temperature. After washing 3 times with TBST for 10 min each, the membranes were exposed to a chemiluminescent substrate (EzWestLumi plus (ATTO, WSE-7120L), and protein bands were identified using the Luminograph II (ATTO, Osaka, Japan) [24]. 

### 2.8. Caspase-3/7 Activity Assay 

KG1 cells were exposed to DLD1-derived CM for 48 h. Caspase-Glo 3/7 assay reagent (Promega, G8090) was added and incubated for 1 h at room temperature. Luminescence was measured by GloMax^TM^ Microplate multimode reader (Promega, Madison, WI, USA) [25]. 

### 2.9. RNA Sequencing Analysis

KG1 cells were treated with DLD1-derived CM for 72 h. The total RNA of the KG1 cells was extracted using TRIzol reagent (Favorgen, FATRR 001). Total RNA quality was measured using an Agilent 2100 Bioanalyzer. QuantSeq 3′ mRNA sequencing was performed through Ebiogen (EBIOGEN, Seoul, Korea). Library construction was performed using a Quentseq 3′ mRNA-Seq Library Prep Kit (Lexogen Inc., Vienna, Austria). Next, single-end 75 bp sequencing was conducted using NextSeq 500 (Illumina, San Diego, CA, USA). Differentially expressed genes (DEGs) were analyzed using ExDEGA (EBIOGEN, Seoul, South Korea). Differentially expressed genes with fold changes ≥ 1.5, normalized data ≥ 4, and *p*-values < 0.05 were used to generate a hierarchal clustering heatmap using ExDEGA Graphic Plus v2.0. The boxplot was created using ggplot2, which is an R script package. To further identify molecular pathways associated with DEGs, we performed an analysis using the Kyoto Encyclopedia of Genes and Genomes (KEGG) database (https://www.genome.jp/kegg/mapper/, accessed on 9 January 2022). The Quantseq raw data were deposited in NCBI and are accessible through accession numbers SPR19118207 and SPR19118208.

### 2.10. Statistical Analysis

All experiments were performed independently at least 3 times to confirm reproducibility. Data are presented as mean ± standard deviation (SD). Statistically significant differences were determined by a two-tailed Mann–Whitney test and a one-way ANOVA test using GraphPad Prism software. Principal component analysis for the normalized mRNA seq data was analyzed by tidyverse R package, and ROC analysis was performed using pROC R package.

## 3. Results

### 3.1. Colorectal Cancer Cell-Derived Conditioned Medium Reduces AML Cell Viability and Increases Apoptosis

**Figure 1:**
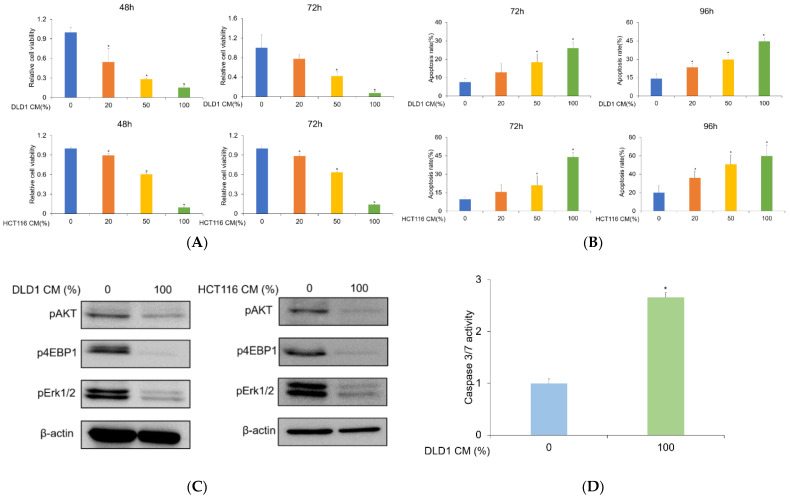
DLD1- and HCT116-derived CM decreases KG1 AML cell viability and induces cell apoptosis. (**A**) KG1 cell viability was measured following treatment with DLD1- or HCT116-derived CM for 48 h or 72 h. An MTS assay was performed to evaluate cell viability. Representative results out of three independent experiments are shown. Statistical significance was carried out using a two-tailed one-way ANOVA test (*n* = 3, * *p* < 0.05). (**B**) KG1 cells were exposed to DLD1- or HCT116-derived CM for 72 h or 96 h. Trypan blue staining was carried out to measure cell apoptosis. The CM increased apoptotic rate dose-dependently (*n* = 3, * *p* < 0.05). (**C**) KG1 cells were treated with DLD1- or HCT116-derived CM for 48 h. The expression of pAKT, pERK1/2, and p4EBP1 was measured by Western blot. β-actin was used as a loading control. The CM reduced phosphorylation levels of AKT, ERK1/2, and 4EBP1. (**D**) KG1 cells were exposed to DLD1-derived CM for 48 h, followed by an analysis of caspase-3/7 activities using ELISA-based bioluminescence assays. The CM increased caspase-3/7 activities (*n* = 3, * *p* < 0.05).

It has been reported that CM obtained from different cell types exhibited various impacts on cellular phenotypes. We aimed to investigate the potential effect of CRC-CM on the survival of AML cells through treatment with DLD1-derived CM in KG1 and HL-60 cells for 48 h and 72 h. To exclude the possibility that growth and survival factors might have been depleted in the CM derived from DLD1 cells, potentially diminishing the survival of the AML cells, we used KG1 CM as a control throughout this study. The cell viability assay demonstrated that DLD1-derived CM decreased KG1 cell viability in a concentration-dependent manner (Figure 1A). Given that the viability of another AML cell line HL-60 was diminished, it is unlikely that the cytotoxic effect of DLD1-derived CM was cell line-specific (Figure S1A). To gain a better idea of how effective the CM was, we compared the cell-killing effect of the chemotherapeutic agent cytarabine with that of CRC-CM (Figure S2A). The IC_50_ values at 72 h for cytarabine and DLD1-CM were 269.8 nM and 44.83%, respectively (Figure S2B). We repeated the same experiment using another colorectal cancer cell line, HCT116, which showed similar results to the DLD1 cells, eliminating the probability of a cell-line-specific effect. To further characterize the effect of CRC-CM on the survival of AML cells, we performed trypan blue staining to measure the apoptotic rates of AML cells. Trypan blue staining results indicated that the apoptotic rate in KG1 cells was significantly increased upon exposure to DLD1- and HCT116-derived CM dose-dependently, as compared to the control (Figure 1B). Also, DLD1-derived CM increased the apoptotic rate of the HL-60 cells (Figure S1B). These data demonstrate that CRC-CM negatively affects cell survival in AML cells.

### 3.2. Colorectal Cancer Cell-Derived Conditioned Media Inhibit PI3K/AKT and MAPK Signaling and Activate Caspases-3/7 in AML Cells

It has been reported that PI3K/AKT/mTOR and MAPK signaling pathways are essential to the proliferation and survival of AML cells. Inhibition of these signaling pathways induces the apoptosis and growth inhibition of these cells. Consequently, we hypothesized that DLD1- and HCT116-derived CM might decrease AML cell survival via downregulation of the PI3K/AKT/mTOR and MAPK pathways. To test our hypothesis, we performed Western blot analysis to identify phosphorylated/activated forms of AKT, 4EBP1, and ERK. DLD1- and HCT116-derived CM reduced the expression of pAKT, p4EBP1, and pERK1/2 (Figure 1C and Figure S1C), suggesting that the anticancer effect of CM in AML cells may be associated with the downmodulation of PI3K/AKT/mTOR and ERK signaling. It has been shown that various stimuli initiate apoptotic pathways, eventually converging to the activation of executioner caspases-3 and -7. We examined whether the CM-induced apoptosis was associated with the activation of caspases-3/7. Treatment of KG1 AML cells with DLD1-derived CM for 48 h resulted in elevation of caspase-3/7 activity (Figure 1D). Our data suggest that CRC-CM decreases the survival rate of AML cells by downregulating the PI3K/AKT and MAPK pathways and increasing caspase-3/7 activities.

### 3.3. Colorectal Cancer Cell-Derived Conditioned Media Induce Myeloid Differentiation

**Figure 2:**
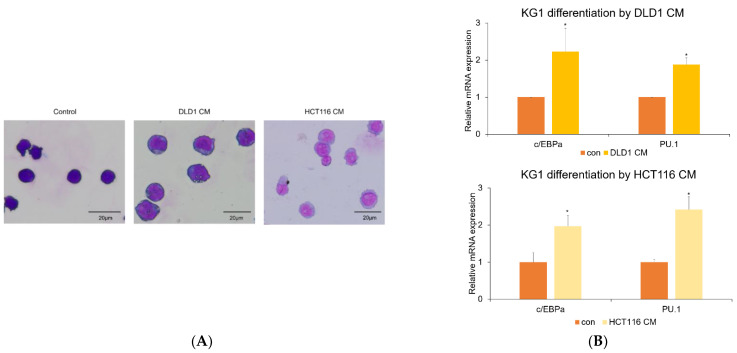
DLD1- and HCT116-derived CM induces KG1 cell differentiation. (**A**) KG1 cells were treated with 20% CRC-CM for 72 h, and Giemsa staining was performed to analyze cell differentiation. (**B**) Relative expression levels of *PU.1* and *c/EBPα* were measured by quantitative real-time PCR (qRT-PCR) following treatment with 20% DLD1- and HCT116-derived CM. The CM increased mRNA levels of *PU.1* and *c/EBPα.* (*n* = 3, * *p* < 0.05).

Next, we examined whether CRC-CM could affect another aspect of AML biology, the differentiation of AML cells. To test this, KG1 AML cells were exposed to DLD1- and HCT116-derived CM for 72 h, followed by Giemsa staining. Giemsa stain analysis revealed that DLD1- and HCT116-derived CM decreased the nucleus size and the nuclear/cytoplasmic ratio, both characteristics of cell differentiation (Figure 2A). DLD1-derived CM also reduced nucleus size and nuclear/cytoplasmic ratio in HL-60 cells (Figure S1D). To attain mechanistic insight into the induction of differentiation of AML cells by CRC-CM, we analyzed the expression levels of transcription factors associated with myeloid differentiation using qRT-PCR following exposure to the CM. As shown in Figure 2B, DLD1- and HCT116-derived CM increased the mRNA levels of *PU.1* and *c/EBPα*, respectively known to be mainly involved in monocytic differentiation and granulocytic differentiation [4]. Also, DLD1-derived CM induces the expression of CD11b, a myeloid differentiation marker (Figure S3).

### 3.4. mRNA-Seq Analysis Revealed That Colorectal Cancer Cell-Derived Conditioned Medium Modulates the Expression of Genes Involved in Apoptosis, Cell Cycle Progression, and Differentiation

To assess statistical separation between cells treated with DLD1-dervied CM and the control group, a principal component analysis (PCA) was conducted (Figure S4). Principal component (PC) 1 (81.42%) had the greatest influence, and PC 2 (11.63%) had the second-greatest influence on the change of gene expression (Figure S4A). The biplot between PC 1 and 2 indicated that genes (*MTRNR2L8, HSP90AB1, RPS3, MALAT1, RPL27A, RPS4X, RPS27A, RPS20, RPS6, TPT1, RPS23, PABPC1, EEF1A1, RLIM, RPL30,* and *RPS29*) have correlations with each main component (Figure S4B). To analyze differential expression between the control and the DLD1-derived CM treated group, the PCA score analysis was conducted. The result identified that the control and DLD1-derived CM-treated group were separated into two clusters in PC 1, showing that the two groups had a difference in gene expression patterns (Figure S4C).

**Figure 3:**
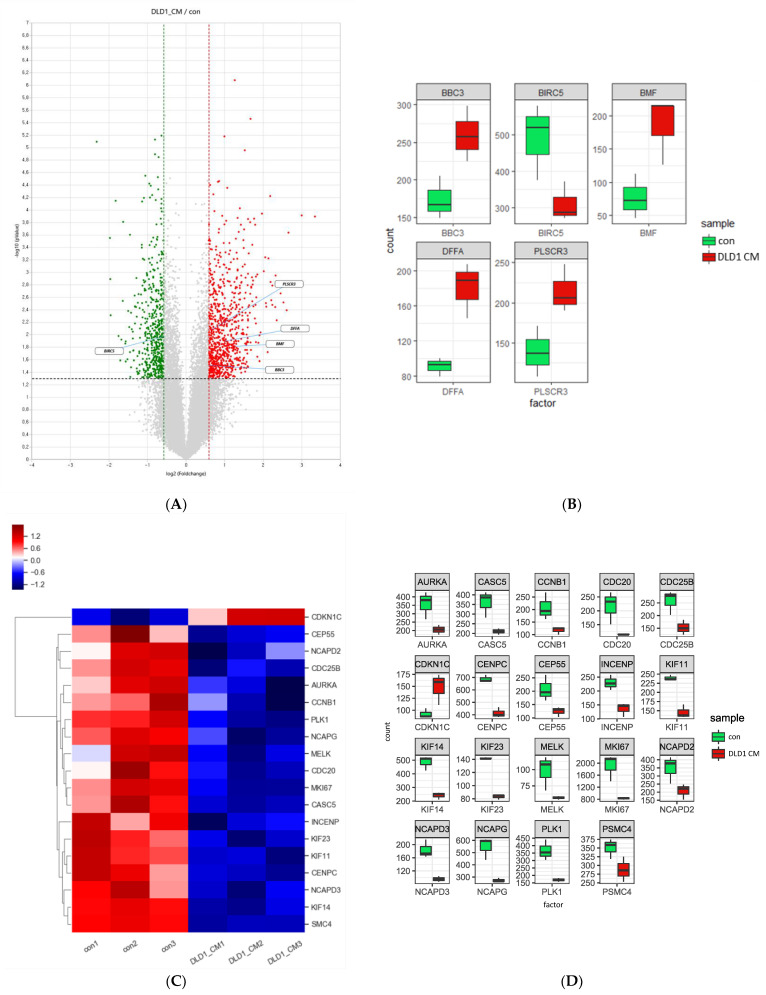
CRC-CM modulates expression of apoptosis- and cell proliferation-related genes. (**A**) Volcano plot of KG1 cells treated with DLD1-derived CM compared to control. (Fold change ≥ 1.5; normalized data ≥ 4; *p* < 0.05). (**B**) Boxplot of apoptosis-related genes in KG1 cells. Green and red boxes indicate gene expression of control and DLD1-derived CM-treated cells, respectively. (**C**) Heatmap of cell cycle regulation-associated genes in KG1 cells. (**D**) Boxplot of cell cycle regulation-associated genes in KG1 cells treated with DLD1-derived CM compared to control.

In previous sections, we demonstrated that the apoptosis and differentiation of AML cells increased upon treatment with CRC-CM. These effects were closely related to the modulation of PI3K/AKT/mTOR and ERK signaling, caspase-3/7 activities, and positive regulators of myeloid differentiation. To identify the genes associated with the phenotypes caused by the CM at the genomic level, we exposed KG1 cells to the CM, followed by QuantSeq 3′ mRNA-sequencing, which identified 280 upregulated genes and 348 downregulated genes. As a result of differentially expressed gene (DEG) analysis, each selected gene list and its raw data of expression related to apoptosis, cell proliferation, and myeloid differentiation are presented in Supplementary Tables S1–S3. Consistent with the phenotype that we observed upon the addition of the CM, expression of apoptosis-related genes, such as *DFFA*, *BMF*, *PLSCR3*, and *BBC3*, increased (Figure 3A,B). Both *BMF* and *BBC3* encoded BH3-only pro-apoptotic proteins of the Bcl-2 family and bound to anti-apoptotic Bcl-2 family members to inactivate them, inducing mitochondrial outer membrane permeabilization and, ultimately, apoptosis [26]. *PLSCR3* encoded phospholipid scramblase 3 protein. Phospholipid scramblase 3 is involved in the redistribution of cardiolipin, a mitochondria-specific phospholipid, resulting in the release of cytochrome c and the activation of a caspase cascade [27,28,29]. The DNA fragmentation factor subunit α encoded by *DFFA* was activated by caspase-3 to induce DNA fragmentation during apoptosis [30]. Conversely, the expression of survival-related genes, such as *BIRC5* and *CLU*, decreased (Figure 3A,B). *BIRC5* belongs to the inhibitor of apoptosis (IAP) gene family and encodes for the BIRC5 protein, also known as survivin. BIRC5/survivin, whose expression is found to be high in most cancers, inhibits caspase activation, preventing cell death [31]. 

Our mRNA-seq analysis also showed that the expression of genes promoting cell cycle progression, such as *CDC20* and *CDC25B*, were downregulated upon treatment with the CM (Figure 3C,D). *CDC20* gene product functions as an activator of anaphase promoting complex [32]. CDC25B, along with CDC25A and CDC25C, eliminates two inhibiting phosphate groups from CDC2, also known as CDK1, thereby promoting mitotic progression [33]. Conversely, expression of *CDKN1C*, a negative regulator of cell proliferation, is upregulated in KG1 cells treated with DLD1-derived CM (Figure 3C,D). These results suggest that CRC-CM modulates many of the genes involved in apoptosis and cell proliferation, which are associated with phenotypes observed in AML cells.

**Figure 4:**
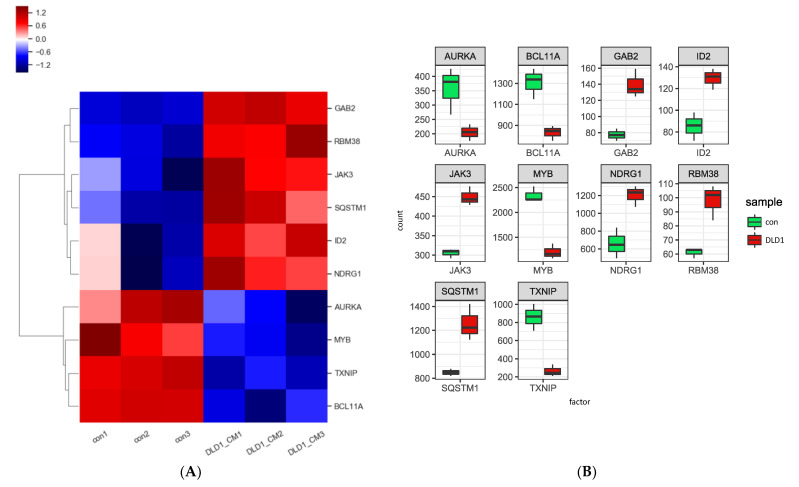

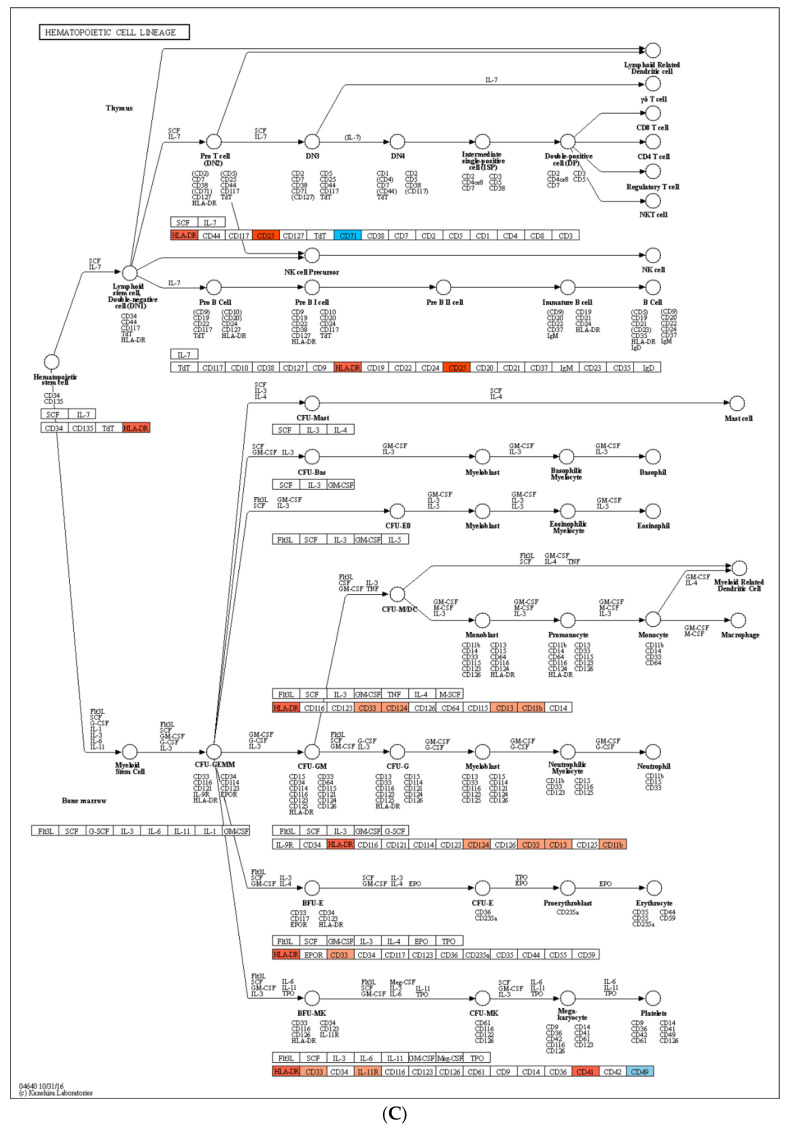
CRC-CM upregulated myeloid differentiation-associated genes. (**A**) Hierarchical clustering heatmap of myeloid differentiation-related genes in KG1 cells treated with DLD1-derived CM versus control. (**B**) Boxplot of myeloid differentiation-related genes in KG1 cells treated with DLD1-derived CM versus control. Green and red boxes indicate gene expression of control and DLD1-derived CM-treated cells, respectively. (**C**) The Kyoto Encyclopedia of Genes and Genomes (KEGG) pathway map for hematopoietic stem cell lineage pathway in KG1 cells treated with DLD1-derived CM. The red color represents upregulated surface markers, while the blue color represents downregulated factors.

Next, we aimed to identify potential genes involved in AML cell differentiation at the genomic level upon exposure to the CM. As shown in Figure 4A,B, the analysis results (heatmap and boxplot) indicated that the expression of differentiation-promoting genes, such as *GAB2, JAK3, ID2, RBM38, SQSTM1,* and *NDRG1* increased, while the expression of *MYB* and *Bcl11A*, myeloid differentiation-inhibiting genes decreased. Based on analysis of the Kyoto Encyclopedia of Genes and Genomes (KEGG) analysis, we found that the surface markers expressed following differentiation into monocytes and granulocytes increased upon exposure to the CM (Figure 4C).

To assess the validity of the gene expression, ROC curves of apoptosis-, cell proliferation- and myeloid differentiation-associated genes were conducted. A larger area under the curve (AUC) correlates with better sensitivity and specificity [34]. Except for *PSMC4* (AUC = 0.889), a cell proliferation-related gene, the results demonstrated that the values of AUC were 1.000, which indicates that the genes have significant abilities for discriminating between the control and DLD1-derived CM-treated groups (Table 1, Table 2 and Table 3, Figure S5). Additionally, Quantseq 3′ mRNA-sequencing results were validated by qRT-PCR. Eight genes (*BIRC5, BMF, MKI67, CDKN1C, PLK1, MYB, NDRG1,* and *RBM38*) in DEGs were selected for validation. The qRT-PCR results were consistent with those of the Quantseq 3′ mRNA-sequencing, suggesting that the DEGs identified by the Quantseq 3′ mRNA-sequencing are valid (Figure S6).

Collectively, these data suggest that CRC-CM exhibits anticancer activities by broadly affecting the expression of genes involved in survival, cell cycle progression, and differentiation in AML cells.

## 4. Discussion

In the present study, we demonstrated that CRC-CM induces apoptosis, cell cycle arrest, and differentiation in AML cells. Rather unexpectedly, our data suggest that colorectal cancer cells secrete molecules with potential anticancer activities in AML cells. These molecules were found to modulate PI3K/AKT and MAPK signaling, which are known to be key survival pathways in AML cells and are central to the expression of differentiation-promoting transcription factors. Our mRNA-seq analysis made it possible to identify genes affected by the CM at the genomic level, revealing that many of survival-, cell cycle progression-, and differentiation-related genes were up- or downregulated. Although this suggests that the mechanisms underlying phenotypes incurred by the CM are quite complex, we are striving to pinpoint key genes in AML cells directly affected by the CM. It would be intriguing to investigate the effect of CM from other types of cancer cells, such as lung and breast cancer, on the survival of AML cells.

The PI3K/AKT and MAPK pathways are shown to be involved in many biological processes, such as cell cycle progression, cell growth and survival, and activation in AML cells. The activation of PI3K/AKT and MAPK signaling is associated with the proliferation of blasts and chemoresistance in AML [35,36]; inhibition of this signaling leads to apoptosis and chemosensitivity, suggesting that these pathways could be therapeutic targets for the treatment of AML. CRC-CM decreased the expression level of the active form of 4EBP1, a well-known substrate of the mTOR signaling pathway. So far, the use of mTOR inhibitors as a monotherapy has shown limited clinical effect in AML patients. In spite of this, combination therapies involving mTOR inhibitors and cytotoxic drugs have shown promising results in improving the complete remission (CR) rates of AML patients [37]. A recent phase Ib/II clinical study on patients with relapsed/refractory AML tested the efficacy of an mTOR inhibitor rapamycin derivative, Everolimus, in combination with a hypomethylating agent, Azacitidine. This drug combination was tolerable and improved overall survival (OS) and overall response rate (ORR) in advanced AML patients [38]. Although the molecules with anticancer activities in the CM have yet to be isolated, it may be worthwhile to examine whether the CM synergize with hypomethylating agents to induce apoptosis in AML cells.

AML cells have the characteristic of differentiation blocking, leading to the accumulation of immature cells and abnormal proliferation. A treatment called “differentiation therapy” promotes the differentiation of these cells into mature cells using a pharmacological agent. For example, all-trans retinoic acid (ATRA) triggers granulocytic differentiation and is currently used as a drug for patients with acute promyelocytic leukemia (APL), a rare subtype of AML. ATRA is highly effective and can induce complete remission in APL. Nonetheless, ATRA has a limitation that makes it ineffective in non-APL subtypes of AML [39,40]. DLD1- and HCT116-derived CM enhance the differentiation of AML cells via the upregulation of *PU.1* and *c/EBPα*, which is known to play a major role in myeloid differentiation. *MYB* proto-oncogene is essential in hematopoiesis and contributes to leukemogenesis by promoting proliferation and suppressing apoptosis. The expression of the *MYB* gene is abundant in immature progenitor cells, and its expression decreases as hematopoietic differentiation progresses [41,42]. Quantseq 3′ mRNA-seq results showed that DLD1-derived CM suppresses *MYB* gene expression. Although it is currently unclear whether cell death, growth arrest, and differentiation are incurred by the same molecule in the CM, these results suggest that DLD1- and HCT116-derived CM have molecules that may be used as a differentiation therapy for AML patients.

Our results in the present study may indicate an inverse correlation between CRC and AML in humans. Instead, previous studies suggest otherwise. Patients with AML aged over 60 may have an increased risk of second primary malignancies, including CRC [43]; therapy-related AML (t-AML) can occur in CRC patients as a direct consequence of mutations induced by various anticancer therapies, such as chemotherapy and radiation therapy [44]; Sawa-Wejksza et al., has demonstrated that, although CRC-CM inhibited proliferation of THP-1 AML cells, it increased expression of vascular endothelial growth factor (VEGF) and matrix metalloproteinase 9 (MMP-9) THP-1 cells, key inducers of angiogenesis and metastasis, respectively [22]. Collectively, these data suggest that application of CRC-CM as a pro-apoptotic agent in AML must be preceded by a deeper understanding of the various factors in CRC-CM, so that we can separate anticarcinogenic factors from procarcinogenic ones.

In conclusion, we demonstrated that CRC-CM unexpectedly showed an anticancer effect on AML cells by triggering cell death and differentiation. Gene expression profiling data supports the notion that this effect is incurred by the extensive modulation of gene expression involved in these biological processes in AML. The isolation of the molecules with anticancer activities from the CM is currently underway and may benefit patients with AML.

## Figures and Tables

**Table 1:** ROC analysis in apoptosis-related genes.

Factor	Individual AUC	Sensitivity (%)	Specificity (%)	95% CI	*p*-Value
*PLSCR3*	1.000	100	100	1.00–1.00	0.0010722
*DFFA*	1.000	100	100	1.00–1.00	0.0010804
*BMF*	1.000	100	100	1.00–1.00	0.0052411
*BBC3*	1.000	100	100	1.00–1.00	0.0028076
*BIRC5*	1.000	100	100	1.00–1.00	0.0003147

**Table 2:** ROC analysis in cell proliferation-related genes.

Factor	Individual AUC	Sensitivity (%)	Specificity (%)	95% CI	*p*-Value
*CDKN1C*	1.000	100	100	1.00–1.00	0.0002743
*CEP55*	1.000	100	100	1.00–1.00	0.0009553
*NCAPD2*	1.000	100	100	1.00–1.00	0.0015402
*CDC25B*	1.000	100	100	1.00–1.00	0.0001392
*AURKA*	1.000	100	100	1.00–1.00	0.0006747
*CCNB1*	1.000	100	100	1.00–1.00	0.0015076
*PLK1*	1.000	100	100	1.00–1.00	0.0000746
*NCAPG*	1.000	100	100	1.00–1.00	0.0002968
*MELK*	1.000	100	100	1.00–1.00	0.0009395
*CDC20*	1.000	100	100	1.00–1.00	0.0010666
*MKI67*	1.000	100	100	1.00–1.00	0.0002308
*CASC5*	1.000	100	100	1.00–1.00	0.0001301
*INCENP*	1.000	100	100	1.00–1.00	0.0002876
*KIF23*	1.000	100	100	1.00–1.00	0.0000944
*KIF11*	1.000	100	100	1.00–1.00	0.0000408
*CENPC*	1.000	100	100	1.00–1.00	0.0000918
*NCAPD3*	1.000	100	100	1.00–1.00	0.0002623
*KIF14*	1.000	100	100	1.00–1.00	0.0000082
*PSMC4*	0.889	100	66.7	0.5809–1.00	0.0014284

**Table 3:** ROC analysis in myeloid differentiation-related genes.

Factor	Individual AUC	Sensitivity (%)	Specificity (%)	95% CI	*p*-Value
*GAB2*	1.000	100	100	1.00–1.00	0.0000009
*RBM38*	1.000	100	100	1.00–1.00	0.0000392
*JAK3*	1.000	100	100	1.00–1.00	0.0004246
*SQSTM1*	1.000	100	100	1.00–1.00	0.0002516
*ID2*	1.000	100	100	1.00–1.00	0.0016399
*NDRG1*	1.000	100	100	1.00–1.00	0.0051907
*AURKA*	1.000	100	100	1.00–1.00	0.0006747
*MYB*	1.000	100	100	1.00–1.00	0.0003980
*TXNIP*	1.000	100	100	1.00–1.00	0.0000531
*BCL11A*	1.000	100	100	1.00–1.00	0.0000216

## Data Availability

Not applicable.

## Supplementary Materials

The following supporting information can be downloaded at: https://www.mdpi.com/article/10.3390/genes13050883/s1: Figure S1: DLD1-derived conditioned medium induces apoptosis and differentiation in HL-60 AML cells; Figure S2: Comparison of cytotoxic effect of DLD1 CM with that of cytarabine; Figure S3: DLD1-derived conditioned medium increases ITGAM expression; Figure S4: principal component analysis (PCA) of mRNA sequencing data; Figure S5: ROC curve analysis for apoptosis-, cell proliferation- and myeloid differentiation-related genes; Figure S6: Validation of Quantseq 3′ mRNA sequencing; Table S1: mRNA seq raw data of apoptosis-related genes which have differential expression after DLD1-derived conditioned medium treatment; Table S2: mRNA seq raw data of cell proliferation-related genes which have differential expression after DLD1-derived conditioned medium treatment; Table S3: mRNA seq raw data of myeloid differentiation-related genes which have differential expression after DLD1-derived conditioned medium treatment.
