# Supplementary material for: Genome-Wide Gene Expression Profiling Defines the Mechanism of Anticancer Effect of Colorectal Cancer Cell-Derived Conditioned Medium on Acute Myeloid Leukemia
Source: Genes (Basel). 2022 May 15;13(5):883. doi: 10.3390/genes13050883 (PMC9171579; doi:10.3390/genes13050883)
Supplement: Supplementary file 1 [file genes-13-00883-s001.zip › genes-1650436 supplementary figures.pdf]

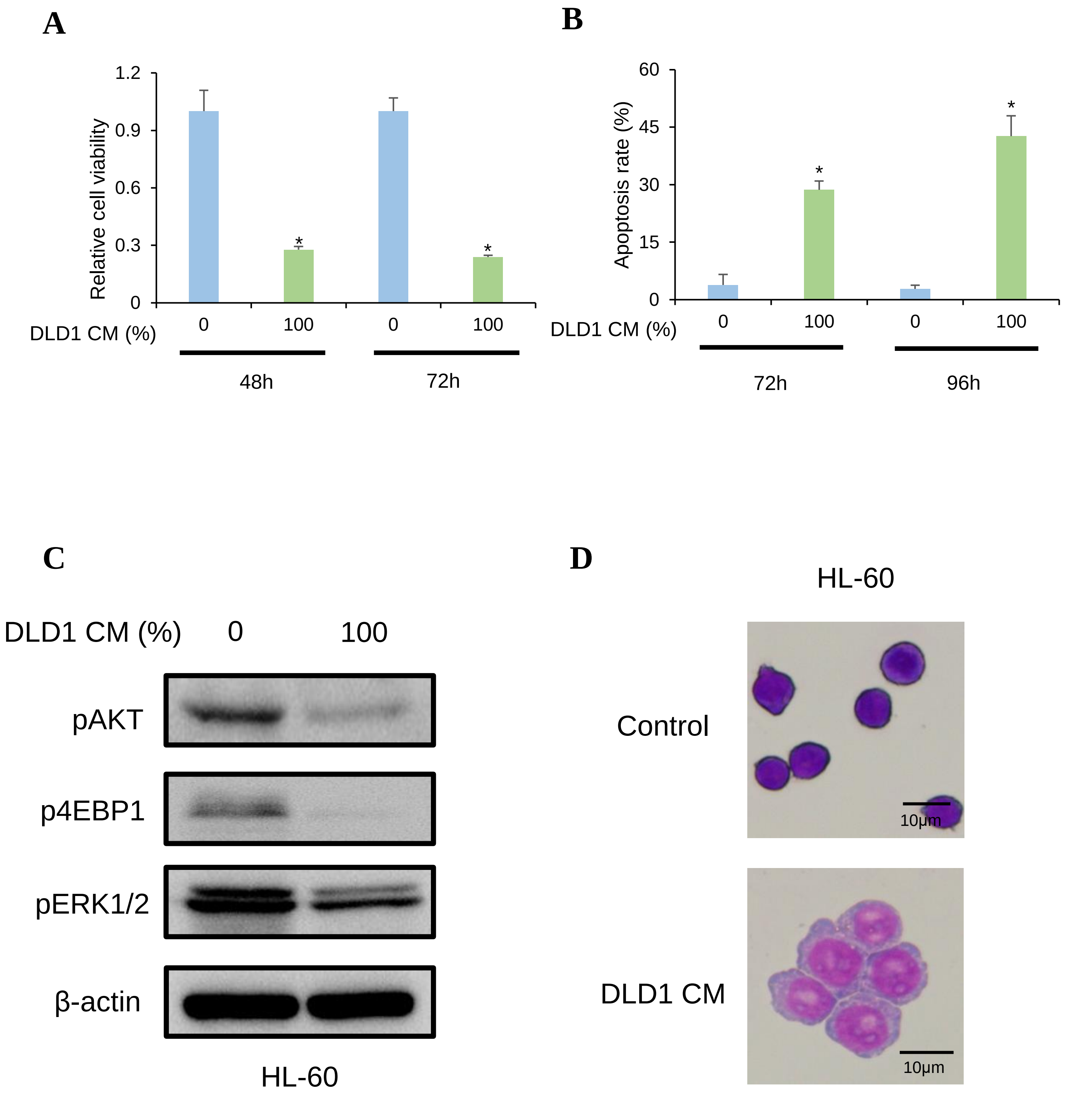

Supplementary figure S1: DLD1-derived conditioned medium induces apoptosis and differentiation in HL-60 AML cells.
(A) HL-60 cells were exposed to DLD1-derived CM for 48 h and 72 h. MTS assays was carried out to measure cell viability. Representative data are shown from three independent experiments (n=3, *p < 0.05).
(B) Cell apoptosis rate was measured after treatment with DLD1-derived CM for 72 h or 96 h. Trypan blue staining was performed to measure cell apoptosis rate (n=3, *p < 0.05).
(C) HL-60 cells were treated with DLD1-derived CM for 48 h. The expression of AKT, ERK and 4EBP1 phosphorylation form was measured by western blot. β-actin was used as loading control.
(D) Giemsa staining was performed after treatment with 20% DLD1-derived CM for 72 h.

A

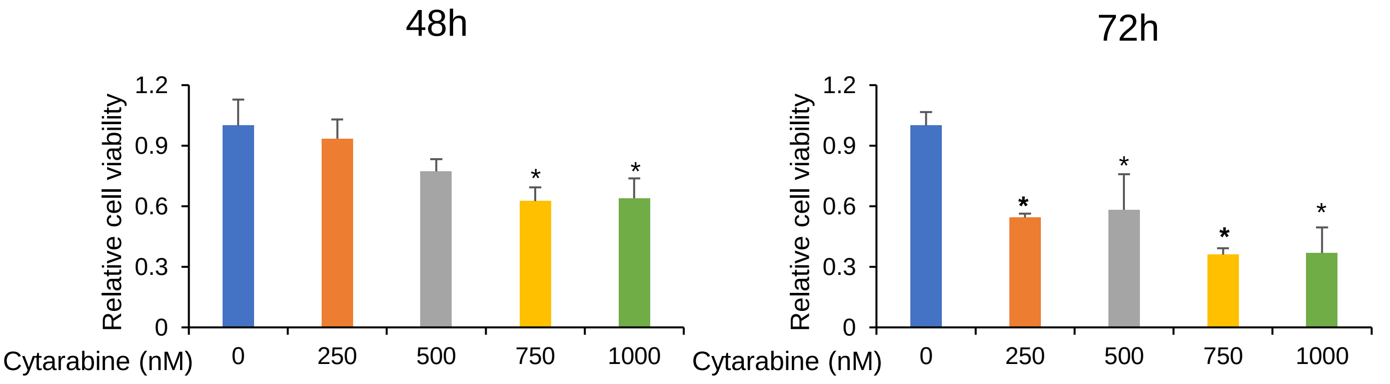

B

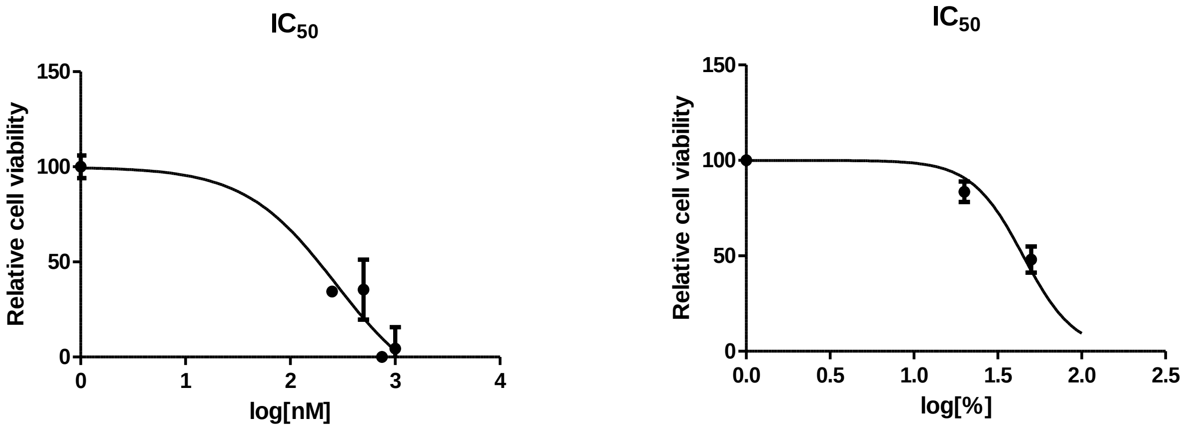

IC_{50} for cytarabine
269.8nM

IC_{50} for DLD1 CM
44.83%

Supplementary figure S2: Comparison of cytotoxic effect of DLD1 CM with that of cytarabine.
(A) KG1 cells were exposed to cytarabine (0, 250, 500, 750 or 1000nM) for 48 h and 72 h. MTS assay was performed to measure cell viability (n=3, *p < 0.05).
(B) IC_{50} values of cytarabine and DLD1-derived CM in KG1 were calculated using GraphPad Prism 5 software.

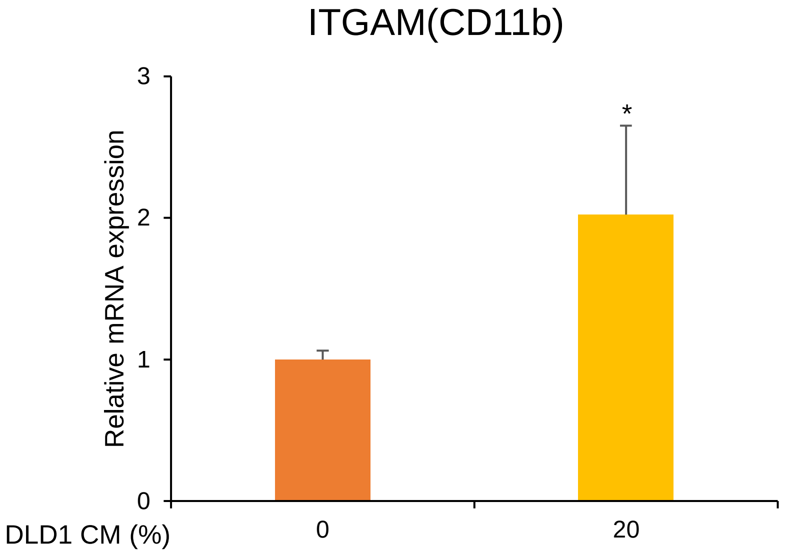

Supplementary figure S3: DLD1-derived conditioned medium increases ITGAM expression.
The ITGAM gene expression was measured by qRT-PCR after KG1 cells were treated with DLD1-derived CM for 72 h (n=3, *p < 0.05).

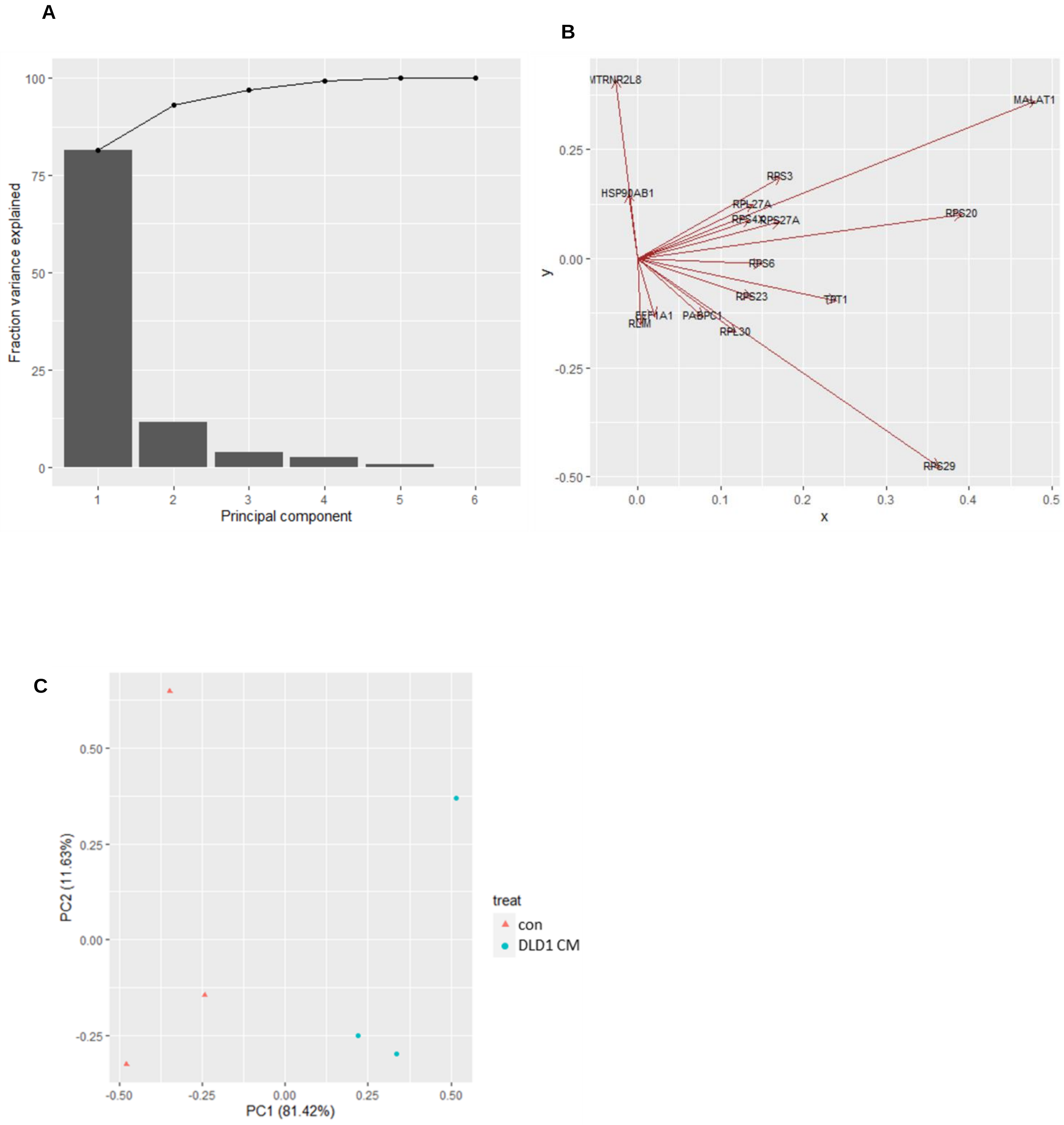

Supplementary figure S4. principal component analysis (PCA) analysis of mRNA sequencing data

(A) PCA scree plot of mRNA sequencing data. the bar chart indicates individual percentage of first 6 PC eigenvalues. Line chart indicate accumulated contribution rate for the first 6 PCs.

(B) Biplot of the genes that have the most influence on each main component and induces differences between groups. The red arrow indicates the PCA of variables in the biplot. The length of the arrows approximates the variance of the variables.

(C) PCA score analysis of mRNA sequencing data. Pink triangles represent the sample treated with DLD-1 CM and blue circles indicate control sample.

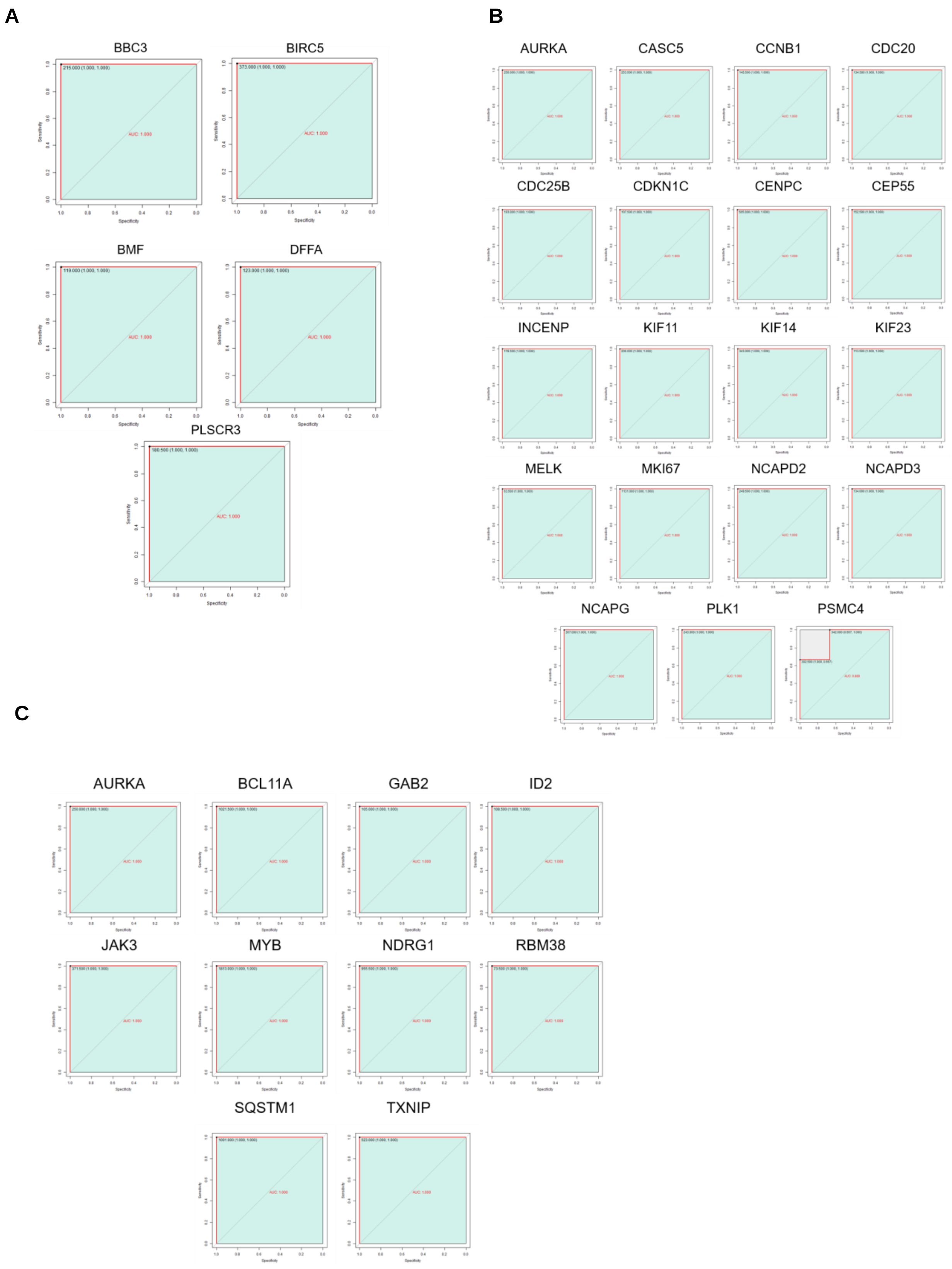

Supplementary figure S5. ROC Curve Analysis for apoptosis-, cell proliferation- and myeloid differentiation-related genes.

(A) The ROC curve of 5 apoptosis associated genes (BBC3, BIRC5, BMF, DFFA and PLSCR3).

(B) The ROC curve of the 19 cell proliferation associated genes (AURKA, CASC5, CCNB1, CDC20, CDC25B, CDKN1C, CENPC, CEP55, INCENP, KIF11, KIF14, KIF23, MELK, MKI67, NCAPD2, NCAPD3, NCAPG, PLK1 and PSMC4).

(C) The ROC curve of 10 myeloid differentiation associated gene (AURKA, BCL11A, GAB2, ID2, JAK3, MYB, NDRG1, RBM38, SQSTM1 and TXNIP).

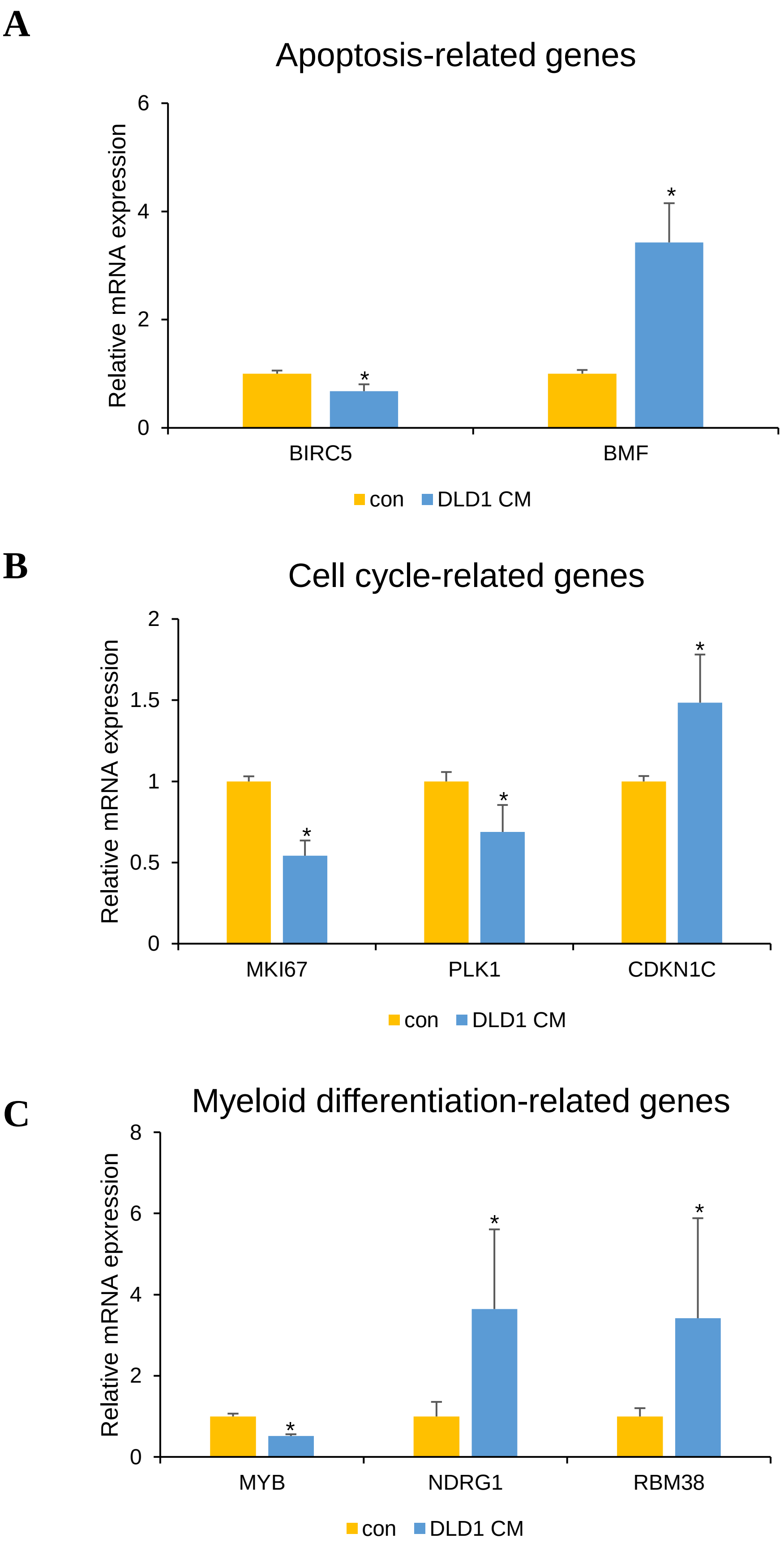

Supplementary figure S6. Validation of Quantseq 3’ mRNA sequencing.
(A) The expression of apoptosis-related genes (BIRC5 and BMF) (B) cell cycle-related genes (MKI67, PLK1 and CDKN1C), and (C) myeloid differentiation-related genes (MYB, NDRG1 and RBM38) was measured by qRT-PCR (n=3, *$p < 0.05$).
