# Supplementary material for: Genome-Wide Gene Expression Profiling Defines the Mechanism of Anticancer Effect of Colorectal Cancer Cell-Derived Conditioned Medium on Acute Myeloid Leukemia
Source: Genes (Basel). 2022 May 15;13(5):883. doi: 10.3390/genes13050883 (PMC9171579; doi:10.3390/genes13050883)
Supplement: Supplementary file 1 [file genes-13-00883-s001.zip › genes-1650436 supplementary tables.pdf]

Table S1. mRNA seq raw data of apoptosis-related genes which have differential expression after DLD1-derived conditioned medium treatment.

Filter: 25737		Fold change	p-value	Average of normalized Data (log2)		Normalized Data (log2)						Raw data						KEGG input	
ID	Gene symbol	DLD1_ CM /con	DLD1_ CM /con	con	DLD1_ CM	con1	con2	con3	DLD1_ CM1	DLD1_ CM2	DLD1_ CM3	con1	con2	con3	DLD1_ CM1	DLD1_ CM2	DLD1_ CM3	Entrez ID	DLD1_ CM /con
9447	PLSCR3	1.581	0.008	4.731	5.392	4.873	4.364	4.896	5.381	5.460	5.332	137	108	171	190	248	206	57048	#FFA07A ,black
168	DFFA	2.001	0.013	4.160	5.161	4.437	3.939	4.057	5.373	5.213	4.850	100	79	93	189	208	146	1676	#FF6347, black
7661	BMF	2.398	0.015	3.909	5.171	3.989	3.224	4.311	4.806	5.252	5.392	72	46	112	126	214	215	90427	#FF6347, black
12125	BBC3	1.509	0.031	5.069	5.663	5.438	4.968	4.704	5.619	5.724	5.644	205	167	149	225	299	257	27113	#FFA07A ,black
10539	BIRC5	0.663	0.011	6.502	5.909	6.294	6.570	6.621	5.888	6.030	5.800	375	518	579	272	371	287	332	#B0E0E6 ,black

Table S2. mRNA seq raw data of cell proliferation-related genes which have differential expression after DLD1-derived conditioned medium treatment.

Filter: 25737		Fold Change	p-value	Average of normalized Data (log2)		Normalized Data (log2)						Raw data						KEGG input	
ID	Gene Symbol	DLD1_ CM /con	DLD1_ CM /con	con	DLD1_ CM	con1	con2	con3	DLD1_ CM1	DLD1_ CM2	DLD1_ CM3	con1	con2	con3	DLD1_ CM1	DLD1_ CM2	DLD1_ CM3	Entrez ID	DLD1_ CM /con
3515	CDKN1C	1.613	0.008	4.172	4.862	4.246	4.053	4.209	4.630	4.963	4.969	87	86	104	111	174	159	1028	#FFA07A ,black
3068	CEP55	0.632	0.036	5.293	4.631	5.142	5.595	5.086	4.540	4.650	4.699	166	261	196	104	139	131	55165	#B0E0E6 ,black
4978	NCAPD2	0.627	0.025	6.013	5.339	5.735	6.121	6.146	5.095	5.298	5.582	253	378	415	155	221	246	9918	#B0E0E6 ,black
14257	CDC25B	0.624	0.004	5.588	4.908	5.424	5.685	5.643	4.795	5.033	4.887	203	278	291	125	183	150	994	#B0E0E6 ,black
14731	AURKA	0.608	0.012	6.051	5.335	5.812	6.132	6.183	5.495	5.372	5.111	267	381	426	206	233	176	6790	#B0E0E6 ,black
18305	CCNB1	0.611	0.027	5.282	4.572	5.107	5.178	5.526	4.838	4.503	4.326	162	194	268	129	125	100	891	#B0E0E6 ,black
8650	PLK1	0.494	0.001	6.089	5.072	6.000	6.027	6.229	5.233	5.018	4.951	305	354	440	171	181	157	5347	#87CEE B,black
17242	NCAPG	0.531	0.003	6.663	5.750	6.525	6.767	6.686	5.993	5.554	5.665	441	595	606	293	265	261	64151	#B0E0E6 ,black
23863	MELK	0.630	0.025	4.239	3.573	3.912	4.364	4.394	3.650	3.445	3.614	68	108	119	54	57	59	9833	#B0E0E6 ,black
687	CDC20	0.572	0.027	5.342	4.536	5.009	5.622	5.329	4.667	4.424	4.507	151	266	233	114	118	114	991	#B0E0E6 ,black
3376	MKI67	0.460	0.004	8.449	7.330	8.179	8.603	8.531	7.490	7.237	7.250	1398	2138	2193	835	864	793	4288	#87CEE B,black
7678	CASC5	0.617	0.006	6.066	5.369	5.889	6.243	6.043	5.410	5.323	5.372	282	412	386	194	225	212	57082	#B0E0E6 ,black
4117	INCENP	0.617	0.007	5.441	4.745	5.582	5.248	5.473	4.579	4.783	4.859	227	204	258	107	153	147	3619	#B0E0E6 ,black
7963	KIF23	0.624	0.003	4.769	4.089	4.913	4.732	4.647	4.163	3.977	4.121	141	141	143	79	85	86	9493	#B0E0E6 ,black
3062	KIF11	0.638	0.002	5.499	4.850	5.619	5.453	5.417	4.881	4.905	4.761	233	236	248	133	167	137	3832	#B0E0E6 ,black
17404	CENPC	0.625	0.004	7.005	6.327	7.130	7.042	6.827	6.375	6.339	6.264	673	721	669	383	461	398	1060	#B0E0E6 ,black
4897	NCAPD3	0.552	0.007	5.129	4.271	5.125	5.322	4.912	4.294	4.128	4.380	164	215	173	87	95	104	23310	#B0E0E6 ,black
2006	KIF14	0.522	0.000	6.498	5.561	6.473	6.550	6.470	5.535	5.477	5.665	425	511	521	212	251	261	9928	#B0E0E6 ,black
11900	PSMC4	0.856	0.098	6.038	5.814	6.064	6.105	5.940	5.959	5.842	5.621	319	374	359	286	325	253	5704	

Table S3. mRNA seq raw data of myeloid differentiation-related genes which have differential expression after DLD1-derived conditioned medium treatment.

Filter: 25737		Fold change	p-value	Average of normalized Data (log2)		Normalized Data (log2)						Raw data						KEGG input	
ID	Gene Symbol	DLD1_ CM /con	DLD1_ CM /con	con	DLD1_ CM	con1	con2	con3	DLD1_ CM1	DLD1_ CM2	DLD1_ CM3	con1	con2	con3	DLD1_ CM1	DLD1_ CM2	DLD1_ CM3	Entrez ID	DLD1_ CM /con
4483	GAB2	1.812	0.000	3.930	4.788	3.951	3.904	3.936	4.795	4.837	4.730	70	77	85	125	159	134	9846	#FFA07A ,black
14744	RBM38	1.610	0.002	3.617	4.304	3.675	3.636	3.536	4.246	4.224	4.432	57	63	63	84	102	108	55544	#FFA07A ,black
11560	JAK3	1.513	0.009	5.852	6.449	6.042	5.839	5.648	6.582	6.385	6.371	314	310	292	443	476	429	3718	#FFA07A ,black
19102	SQSTM1	1.528	0.007	7.306	7.917	7.461	7.225	7.218	8.038	7.951	7.749	848	819	879	1223	1421	1123	8878	#FFA07A ,black
12657	ID2	1.526	0.022	4.081	4.691	4.409	3.814	3.951	4.726	4.568	4.771	98	72	86	119	131	138	3398	#FFA07A ,black
23515	NDRG1	1.869	0.034	6.964	7.866	7.444	6.505	6.777	8.126	7.749	7.683	838	495	646	1301	1234	1073	10397	#FFA07A ,black
14731	AURKA	0.608	0.012	6.051	5.335	5.812	6.132	6.183	5.495	5.372	5.111	267	381	426	206	233	176	6790	#B0E0E6 ,black
20193	MYB	0.529	0.010	8.774	7.856	9.029	8.679	8.575	7.964	7.901	7.690	2523	2254	2261	1162	1372	1078	4602	#B0E0E6 ,black
1329	TXNIP	0.322	0.000	7.308	5.673	7.204	7.305	7.409	5.522	5.894	5.576	709	866	1004	210	337	245	10628	#87CEE B,black
12980	BCL11A	0.664	0.000	7.917	7.327	7.899	7.929	7.924	7.343	7.209	7.420	1150	1338	1438	754	847	893	53335	#B0E0E6 ,black
